# Noncovalent functionalization of carbon nanotubes as a scaffold for tissue engineering

**DOI:** 10.1038/s41598-022-16247-7

**Published:** 2022-07-14

**Authors:** Mohyeddin Assali, Naim Kittana, Sahar Alhaj-Qasem, Muna Hajjyahya, Hanood Abu-Rass, Walhan Alshaer, Rula Al-Buqain

**Affiliations:** 1 Department of Pharmacy, Faculty of Medicine & Health Sciences, An-Najah National University, Nablus, Palestine; 2 Department of Biomedical Sciences, Faculty of Medicine & Health Sciences, An-Najah National University, Nablus, Palestine; 3 Department of Physics, Faculty of Sciences, An-Najah National University, Nablus, Palestine; 4 Cell Therapy Center, The University of Jordan, Amman, 11942 Jordan

**Keywords:** Materials chemistry, Nanomedicine, Tissue engineering

## Abstract

Tissue engineering is one of the hot topics in recent research that needs special requirements. It depends on the development of scaffolds that allow tissue formation with certain characteristics, carbon nanotubes (CNTs)-collagen composite attracted the attention of the researchers with this respect. However, CNTs suffer from low water dispersibility, which hampered their utilization. Therefore, we aim to functionalize CNTs non-covalently with pyrene moiety using an appropriate hydrophilic linker derivatized from polyethylene glycol (PEG) terminated with hydroxyl or carboxyl group to disperse them in water. The functionalization of the CNTs is successfully confirmed by TEM, absorption spectroscopy, TGA, and zeta potential analysis. 3T3 cells-based engineered connective tissues (ECTs) are generated with different concentrations of the functionalized CNTs (*f*-CNTs). These tissues show a significant enhancement in electrical conductivity at a concentration of 0.025%, however, the cell viability is reduced by about 10 to 20%. All ECTs containing *f*-CNTs show a significant reduction in tissue fibrosis and matrix porosity relative to the control tissues. Taken together, the developed constructs show great potential for further in vivo studies as engineered tissue.

## Introduction

Regenerative medicine is a relatively new multidisciplinary field of medical science that aims to regenerate cells, tissues and organs to restore, preserve or reinforce their functions^1,2^. It is envisioned that none curable diseases, like Alzheimer's disease, Parkinson's disease, spine injuries, and heart failure could be treated in the future with such an approach^3–5^. Tissue engineering is the main approach in regenerative medicine that is based mainly on designing a three-dimensional (3D) biocompatible scaffold that supports the growth of the cells^6^. The constituents and the design of the tissue’s scaffold should mimic the natural extracellular matrix (ECM) of the original tissue^7^, which constitutes generally proteoglycans, adhesion proteins, and networks of collagen and elastin fibers^8–10^.

The interest in applying nanotechnology in regenerative medicine is rising, as it provides solutions to generate scaffolds with nanostructures that are more capable to mimic natural tissues^11^. Recently, carbon nanotubes (CNTs) gained a special interest in this respect because of their unique electrical and mechanical properties. The electrical current density through CNTs can reach up to 1,000 times greater than that through copper wires^12^. In addition, their tensile strength can be up to 63 gigapascals (GPa), which is around 50 times greater than that of steel^13^. At the same time, their elastic modulus value could be between 1.0 and 1.8 terapascal^14^. The utilization of the unique CNT’s physicochemical properties in the construction of tissues requires the organization of CNTs in the form of a 3D configuration. It has been shown formerly that the multi-walled carbon nanotubes (MWCNTs) cross-linked into a porous 3D scaffold formed cytocompatible surfaces for the growth of cells^15,16^. CNTs come in two forms; single-walled (SWCNTs) and multi-walled (MWCNTs), depending on the number of the graphite layers that fold to form the nanotubes^17^. The diameter of the SWCNTs is in the range of 0.4–2 nm while the diameter of the MWCNTs is in the range of 2–30 nm with various lengths up to 10 µm^18^. Depending on their chirality, SWCNTs can be classified as metallic or semiconducting nanomaterials^19^. The metallicity and semiconductivity of the MWCNTs depend on the electrical transport properties, the outer diameter, and the intershell interaction^20^. SWCNTs tend to form huge intact bundles with various lengths due to the Van Der Waals force between the nanotubes making them more difficult to obtain individual nanotubes or to be functionalized^21^. However, in the case of MWCNTs individual nanotubes can be easily resolved^20^.

Despite the interesting physicochemical properties of CNTs, they are not free of toxicity, which has been investigated by many researchers. The toxicity was mainly mediated by oxidative stress and inflammatory reactions^22^. Moreover, the poor solubility of CNTs in most organic solvents and especially the aqueous solutions adversely affects their use in biological applications^23^. Therefore, suitable and proper functionalization (covalently or non-covalently) on the surface of the CNTs with adequate moieties can improve their water dispersibility, and biocompatibility and thus decrease their toxicity^24,25^. The non-covalent functionalization, in contrast to the covalent functionalization, can maintain the sp^2^ nanotube structure, π-conjugated structure and so the electronic characteristics and other optical properties of CNTs^23,26–28^, which makes them more favorable for tissue engineering applications, especially where efficient electrical conductivity is required as in the case of cardiac and neural tissue engineering, where the poor electrical conductivity of the connective tissue is believed to challenge the electrical integration of the implanted engineered tissues^29–32^. Moreover, it has been demonstrated by several research groups that in vitro electrical stimulation of cells can enhance the optimization of the cultured cells as demonstrated by enhancing neurite outgrowth from neurons, and also by inducing the differentiation of osteoblasts and by increasing the deposition of collagen by these cells^33^. Therefore, it is believed that there is a need to develop conductive biomaterials that could facilitate the electrical stimulation of the cells within the engineered tissues.

It was previously shown that the pyrene moiety can interact effectively on the surface of the CNTs through π-π stacking and depending on the functionalization process could produce *f*-CNTs with good water dispersibility and biocompatibility^23,34^. The functionalized carbon nanotubes (*f*-CNTs) can be combined with polymeric hydrogels, such as collagen, to form novel porous three-dimensional nanostructure scaffolds that combine the properties of both collagen and functionalized CNTs^35,36^.

**Figure 1:**
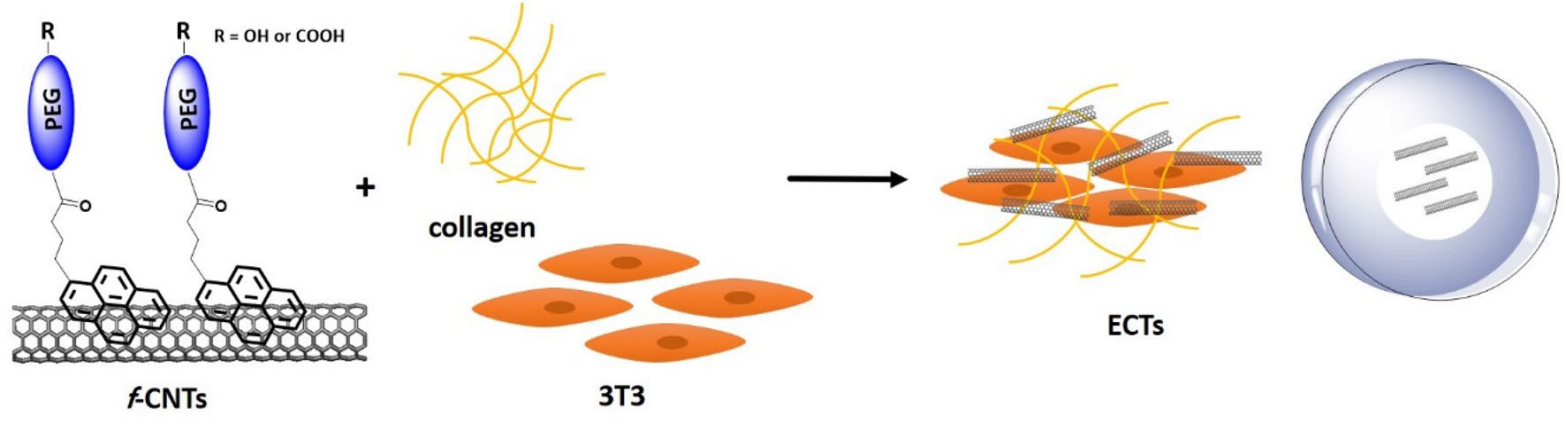
Schematic representation of the engineered connective tissue based on the non-covalent functionalization of the CNTs.

Herein, we aim to functionalize SWCNTs and MWCNTs non-covalently with synthesized pyrene-polyethylene glycol derivatives that are terminated with hydroxyl or carboxyl group and then to study the impact of incorporating them in collagen-based engineered connective tissues on specific physical and structural properties of these tissues as shown in Fig. 1.

## Materials and methods

### Reagents and instrumentations

All reagents and materials utilized in the experiments were of analytical grade. The l-ascorbic acid sodium salt, 1-(3-Dimethylaminopropyl)-3-ethylcarbodiimide hydrochloride (EDC), propargyl bromide, tetraethylene glycol (TEG), and 1-Pyrenebutyric acid were purchased from (Alfa Aesar company, England). Sodium azide was purchased from (RiedeldeHaёn Company, Germany). 4-(dimethylamino) pyridine (DMAP), tetrahydrofuran anhydrous, and anhydrous copper sulfate were purchased from (Sigma-Aldrich, Germany). Short SWCNTs (diameter: 1–2 nm, length: 1–3 µm, purity > 90%, and batch number: 1246YJS) and MWCNTs (diameter: 8–15 nm, length: 0.5–2 µm, purity > 95%, and batch number: 1235YJS) were purchased from (Nanostructured & Amorphous Materials, Inc USA). All reactions were stirred beneath ambient conditions. Column chromatography utilizing silica gel (pore size 60 Å) purchased from (Sigma Aldrich Company) was utilized to purify the products. Dulbecco’s Ca^++^ -free phosphate-buffered saline, DMEM, and l-glutamine solution were purchased from (Biological industries, Jerusalem). Trypsin–EDTA solution 0.025, fetal bovine serum, trypan blue solution, DMEM powder, and collagen solution from bovine skin were purchased from (Sigma-Aldrich, Germany). Cell titer 96 Aqueous one solution cell proliferation Assay was purchased from (Promega, USA). The 3T3 fibroblast cell line from the American Type Culture Collection (ATCC), Manassas, VA, USA) was obtained as a kind gift from Dr. Johnny Amer.

Ultraviolet–Visible (UV–VIS) spectra were measured using 10-mm quartz cuvettes in (7315 Spectrophotometer, Jenway, UK). Water bath sonicator (MRC DC-200H Digital Ultrasonic Cleaner) was utilized in the preparation and dispersion of functionalized CNTs. Dynamic light scattering and zeta potential were measured in NanoBrook Omni (Brookhaven Instruments, USA). Electrical conductivity was captured utilizing Four Probe Method AL-212 (Acumen labware, Ambala, India). Fourier-transform infrared spectroscopy (FTIR) was done on Nicolet iS5 (Thermo Fisher Scientific Company, USA). Nuclear Magnetic Resonance (NMR) spectra were obtained using Bruker Avance (500 spectrometers, Switzerland). Thermogravimetric analysis (TGA) spectra were recorded in the range 25–600 °C, flow rate 20 °C under nitrogen (100 cc min^−1^) by (STA 409 PC luxx, NETZSCH). Transition electron microscope (TEM) images were taken at 60 kV using Morgagni 286 transmission microscope (FEI Company, Eindhoven, Netherlands). Scanning electron microscope (SEM) images were done on Versa 3D (FEI Company, Eindhoven, Netherlands). Digital microscope images for histopathological evaluation were done on Leica ICC50 HD (Leica camera AG company, Wetzler, Germany). Unilab microplate reader 6000 was utilized in the cell viability test to read the plate.

### Synthesis and characterization

All the synthetic procedures were conducted at An-Najah University labs. NMR, SEM, TEM, and TGA measurements were run at the University of Jordan.

#### Synthesis of OH-TEG-alkyne (1)

TEG (3 g, 15.4 mmol) was dried under vacuum and dissolved in anhydrous THF (20 mL) under argon. In another round bottom flask, sodium hydride (NaH) (741 mg, 30.9 mmol) was dried under vacuum and dissolved in anhydrous THF (10 mL) under argon. NaH solution was added to the TEG solution dropwise until the hydrogen gas (H_2_) was released. After that, propargyl bromide (1.9 mL, 21.6 mmol) was dried under vacuum and dissolved in anhydrous THF (10 mL) under argon and was added to the reaction. The reaction was vigorously stirred overnight at room temperature. The next day, water (H_2_O) (5 mL) was added to the reaction drop by drop, and the reaction was evaporated. The crude product was extracted by DCM (100 mL) then it was dried by Na_2_SO_4_, filtered, and evaporated. The product was purified by silica column chromatography in ethyl acetate to obtain an oily yellow product with a yield of (1.7 g, 7.3 mmol, and 47.4%). *R*_*f*_*:* 0.46 (Ethyl acetate). ^1^H NMR and ^13^C NMR were obtained as in the literature^37^.

#### Synthesis of pyrene-TEG-alkyne (2)

Compound (1) (1 g, 4.3 mmol), pyrenebutyric acid (620.7 mg, 2.2 mmol), 1-Ethyl-3-(3-dimethylaminopropyl) carbodiimide hydrochloride (EDC) (1.2 g, 6.5 mmol) and 4-Dimethylaminopyridine (DMAP) (396.2 mg, 3.2 mmol) were dissolved in DCM (20 mL) and were reacted under argon**.** The reaction was vigorously stirred for 24 h at room temperature. The crude product was extracted by DCM (170 mL) and 1 M HCL (50 mL) then it was dried by Na_2_SO_4_, filtered and evaporated. The product was purified by silica column chromatography in ethyl acetate/n-hexane (1:2) to obtain an oily yellow product with a yield of (980 mg, 1.9 mmol, 44.2%). R_*f*_: 0.57 (Ethyl acetate/n-Hexane (1:1)). ^1^H NMR: (500 MHz, CDCl_3_): δ 8.29–7.83 (m, 9H, Py), 4.23 (s, 2H, CH_2_OCO), 4.15 (s, 2H, OCH_2_C≡CH), 3.67–3.53 (m, 14H, 7CH_2_O), 3.37 (t, 2H, *J* = 7.2 Hz, Py-CH_2_), 2.47 (t, 2H, *J* = 7.2 Hz, CH_2_COO), 2.39 (s, 1H, C≡CH), 2.20–2.15 (quint, 2H, Py-CH_2_CH_2_). ^13^C NMR (125.7 MHz, CDCl_3_): δ 173.5, 135.8, 131.4, 130.9, 130.0, 128.7, 127.5, 127.4, 126.7, 125.9, 125.1, 125.0, 124.9. 124.8, 123.4, 72.5, 70.5, 70.4, 70.2, 69.7, 69.2, 69.1, 64.6, 63.5, 61.6, 61.5, 61.0, 33.8, 32.7, 29.7, 26.8.

#### Synthesis of OH-TEG-Tosyl (3), OH-TEG-N_3_ (4), and COOH-TEG-N_3_ (5)

These compounds were synthesized and identified as in the literature^38,39^.

#### Synthesis of Pyrene-TEG-triazole-TEG-OH (6)

Compound (2) (296.5 mg, 0.6 mmol) and compound (4) (194 mg, 0.9 mmol) were dissolved in DCM (8 mL) and a solution of sodium ascorbate (233.7 mg, 1.2 mmol) and anhydrous copper sulfate (188.3 mg, 1.2 mmol) in H_2_O (8 mL) was added. The reaction was vigorously stirred for 24 h at room temperature. The product was extracted by DCM (160 mL) and H_2_O (50 mL) then it was dried by Na_2_SO_4_, filtered and evaporated. The product was purified by silica column chromatography in DCM/MeOH (20:1) to obtain an oily yellow product with a yield of (260 mg, 0.4 mmol, 66.7%). R_*f*_: 0.57 (DCM/MeOH (20:1)). ^1^H NMR: (500 MHz, CDCl_3_): δ 8.22–7.78 (m, 9H, Py), 7.68 (s, 1H, CH triazole), 4.62 (s, 2H, CH_2_-triazole), 4.39 (t, 2H, *J* = 4.9 Hz, CH_2_OCO), 4.20 (t, 2H, *J* = 4.8 Hz, CH_2_N), 3.69 (t, 2H, *J* = 4.9 Hz, CH_2_OH), 3.65–3.42 (m, 26 H, 13CH_2_O), 3.32 (t, 2H, *J* = 7.6 Hz, CH_2_CO), 2.43 (t, 2H, *J* = 7.4 Hz, Py-CH_2_), 2.13 (quint, 2H, Py-CH_2_CH_2_). ^13^C NMR (125.7 MHz, CDCl_3_): δ 173.4, 135.7, 131.4, 130.9, 130.0, 128.7, 127.5, 127.4, 126.7, 125.9, 125.1, 125.0, 124.9, 124.8, 124.0, 123.3, 72.7, 70.5, 70.4, 70.2, 69.6, 69.3, 69.1, 64.5, 63.5, 61.6, 50.2, 33.8, 32.7, 29.7, 26.8. HRMS (ESI, m/z): calculated for C_39_H_52_N_3_O_10_ [M + H]^+^ 722.3653, found 722.3651.

#### Synthesis of Pyrene-TEG-triazole-TEG-COOH (7)

To a solution of compound (2) (287.3 mg, 0.6 mmol) and compound (5) (200 mg, 0.9 mmol) in DCM (8 mL) a solution of sodium ascorbate (226.5 mg, 1.1 mmol) and anhydrous copper sulfate (182.5 mg, 1.1 mmol) in H_2_O (8 mL) was added. The reaction was vigorously stirred for 24 h at room temperature. The product was extracted by DCM (160 mL) and H_2_O (50 mL) then it was dried by Na_2_SO_4_, filtered and evaporated. The product was purified by silica column chromatography in DCM/MeOH (20:1) to obtain an oily yellow product with a yield of (180 mg, 0.2 mmol, 33.3%). R_*f*_: 0.49 (DCM/MeOH (20:1)). ^1^H NMR: (500 MHz, CDCl_3_): δ 8.29–7.83 (m, 9H, Py), 7.76 (bs, 1H, triazole), 4.63 (s, 2H, CH_2_COOH), 4.58 (s, 2H, CH2-triazole), 4.49 (t, 2H, *J* = 5.0 Hz, CH_2_OCO), 4.22 (t, 2H, *J* = 4.8 Hz, CH_2_N), 3.82 (t, 2H, *J* = 4.9 Hz, triazole-CH_2_CH_2_), 3.68–3.55 (m, 22H, 11CH_2_O), 3.37 (t, 2H, *J* = 7.7 Hz, CH_2_CO), 2.47 (t, 2H, *J* = 7.3 Hz, Py-CH_2_), 2.11 (quint, 2H, Py-CH_2_CH_2_). ^13^C NMR (125.7 MHz, CDCl_3_): δ 173.5, 170.4, 135.8, 131.4, 130.9, 130.0, 128.7, 127.5, 127.4, 126.7, 125.9, 125.1, 125.0, 124.9, 124.8, 123.4, 70.9, 70.5, 70.4, 69.7, 69.3, 69.2, 69.0, 68.5, 64.6, 63.7, 63.6, 63.5, 53.5, 33.8, 32.8, 29.7, 26.8. HRMS (ESI, m/z): calculated for C_39_H_50_N_3_O_11_ [M + H]^+^ 736.3445, found 736.3443.

### Optimization of the needed amount of compound (6) or (7) to functionalize SWCNTs and MWCNTs in H_2_O

Different quantities of compound (6) or (7) (0.25, 0.5, 1 mg) were dissolved in H_2_O (1 mL) and were added to different eppendorfs containing 1 mg of SWCNTs or MWCNTs and the solutions were sonicated in water bath sonicator in a continuous mode and controlled temperature for 30 min to reach a concentration of 1 mg ml^−1^ of the SWCNTs or MWCNTs followed by a centrifugation process and washing steps with distilled water to remove the excess of the pyrene derivatives. Finally, the different dispersions were preserved under observation to study the optimum concentration needed to disperse the CNTs in water without the formation of any precipitation or excess micellar structures as confirmed by TEM images.

### Sample preparation for TEM analysis

5 µl of the sample was dropped on a carbon-coated copper grid of 200 mesh (Ted Pella Inc.), then incubated for 10 s and the excess of the liquid was blotted away using filter paper. Finally, the grid was left to dry in the air.

### Generation of engineered connective tissues (ECTs)

3T3 fibroblast cells were cultured and maintained in high-glucose DMEM supplied with 15% FBS and under standard cell culture conditions. We followed a method for ECT generation that was described in recent publications by our group^40,41^, which was a modified method from Tiburcy and colleagues^42^. In brief, 10 × DMEM was prepared from DMEM powder that was further used to prepare 2X DMEM by diluting the original stock in sterile water and providing FBS up to 30%. Solutions containing the different types of functionalized CNTs (prepared in PBS) were mixed with 2X DMEM with ratios that achieve the required final concentrations in the ECT (0.025%, 0.050, and 0.1% of *f*-CNTs). Then acid-soluble collagen solution was added, and the mixture was quickly neutralized by 0.1 N NaOH before the cell suspension was added immediately and mixed thoroughly. The mixture was cast in 48-well plates pre-coated with gelatin. After the tissues were condensed, the growth medium was added. Each 200 µL-size ECT contained 2.5 × 10^6^ cells of 3T3 cells.

### Measuring cell viability by MTS test

The CNTs test compounds were sterilized by irradiation with UV light for 10 min. After that, they were suspended in a culture medium and sonicated for 90 min to disintegrate the aggregates and to well disperse the particles in the medium.

The MTS test was carried out according to the manufacturer’s protocol. In brief, 3T3 cells seeded in a 96-well plate were incubated with a serial dilution of the test solutions (0.025%, 0.050, 0.1% of *f*-CNTs, and 0.0% as control) overnight. The experiment included wells with growth medium but without cells to serve as blank samples. Also, some wells contained cells but did not receive any of the test compounds to serve as a negative control. After incubating the wells with the treatment conditions for 24 h, the supernatant was removed and the wells were washed carefully three times with PBS to eliminate the CNTs from the wells, then 100 µL of fresh medium containing 10% MTS reagent was added to each well, and the plate was incubated for 1 h in a cell culture incubator. Next, the supernatant was transferred to a fresh 96-well plate and the absorbance of the supernatant was measured at a wavelength of 492 nm.

### Measuring the electrical conductivity of ECTs

The four-probe method was used to measure the electrical conductivity of the developed engineered tissue with the various concentrations of the *f*-CNTs (0.025%, 0.050, 0.1% of *f*-CNTs, and 0.0% as control), which was calculated from the voltage measurements along with variable electrical current values^43^. This method includes driving four evenly spaced probes in connection with the center of material with unknown conductivity. The two outer probes are utilized for exporting current and the two inner probes are utilized for measuring the outcoming voltage drop across the surface of the material^44^. The conductivity (σ) can be calculated as:

$$\upsigma = 1/\uprho $$

where ρ is the resistivity, and it can be calculated as:

$$\uprho =\uprho ./[\mathrm{G}7 (\mathrm{W}/\mathrm{S})]$$

where G7 is a correction factor, which is a function of the material thickness (W) and the distance between probes (S). The volume resistivity (ρ.) can be calculated as:

$$\uprho . =\mathrm{ V x }2\mathrm{ \pi S}/\mathrm{I}$$

where (V) is the measured voltage, (S) is the distance between probes, and (I) is the source current^45^.

### Microscopic evaluation for ECTs

All tissue samples were fixed in 4% paraformaldehyde (PFA) overnight. Then they were sectioned, processed, and stained at the medical laboratory at An-Najah University Hospital according to the standard routine protocol followed for pathological samples. The tissue samples were stained with Masson’s trichrome stain and then they were examined by an independent-blinded clinical pathologist. In addition, the grade of fibrosis in the stained tissues was further analyzed digitally by ImageJ software loaded with a “color deconvolution” plug-in.

For scanning electron microscopy (SEM), some of the fixed ECTs were frozen by liquid nitrogen, and then they were fractured cryogenically using the surgical operation blade. The sections were dried on a stub before they were imaged^46^. The SEM images were analyzed by ImageJ software to investigate the average diameter of the pores (matrix porosity) and the effect of CNTs compounds on the interaction between the collagen fibers.

### Statistical evaluation for ECTs

The statistical analysis for electrical conductivity, cell viability, histopathological evaluation, and SEM evaluation and the graphs were prepared by GraphPad Prism software (GraphPad Software, La Jolla, CA, USA). A two-way ANOVA test and Student’s t-test were used to compare the means.

## Results and discussion

### Synthesis of pyrene conjugates and functionalization of CNTs

**Figure 2:**
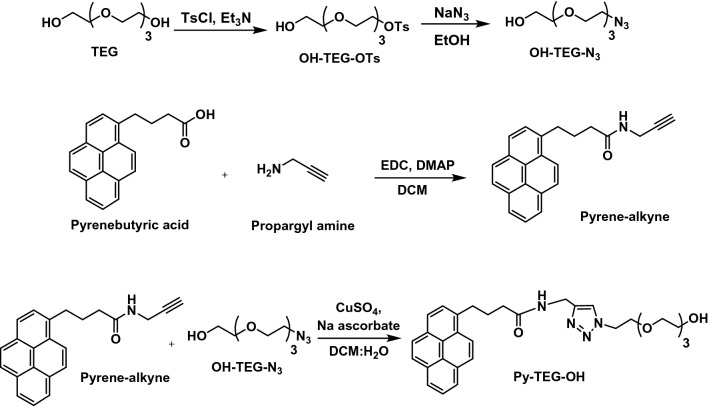
Synthesis approach of the pyrene-TEG-OH.

In this project, we aim to functionalize the CNTs (SWCNTs, MWCNTs) non-covalently to preserve the electronic properties of the carbon nanostructure to be a scaffold for tissue engineering. For this purpose, we utilized the pyrene moiety to interact with the surface of the carbon nanostructures through π-π stacking^47^. Moreover, the pyrene moiety was connected to a hydrophilic linker to obtain the adequate hydrophobic/hydrophilic balance that capable to disperse the carbon nanostructures in water. A derivative of polyethylene glycol (PEG) was selected to be connected to the pyrene moiety. PEG is an FDA approved non-toxic and biodegradable polymer that has the ability to enhance the water dispersibility and stability and improve the biocompatibility of the conjugated CNTs^48–50^. Therefore, in the first place, the hydrophilic linker was synthesized based on the tetraethylene glycol (TEG) derivative. The synthesis was started by the selective mono-tosylation of TEG using tosyl chloride followed by its reaction with sodium azide to form the OH-TEG-N_3_. After that, the pyrene butyric acid was reacted with propargyl amine to form py-alkyne. In a final step, a click reaction was performed between the Py-alkyne and the linker OH-TEG-N_3_ using copper sulfate and sodium ascorbate as a catalyst to produce the Py-TEG-OH as shown in Fig. 2.

**Figure 3:**
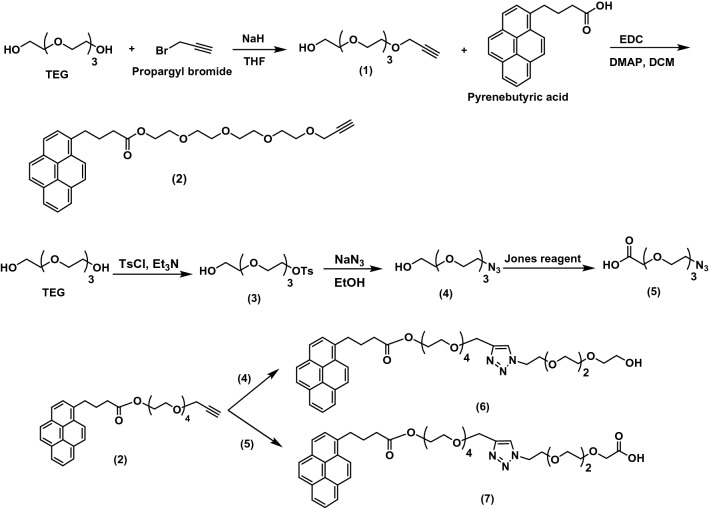
Synthesis of Py-TEG-triazole-TEG-OH (6) and Py-TEG-triazole-TEG-COOH (7).

Once the compound was successfully synthesized, it was tested to disperse the carbon nanotubes in water. Unfortunately, the synthesized compound was incapable to disperse the CNTs effectively in water supposing that the length of the hydrophilic linker is not sufficient. For that reason, we decided to synthesize a double linker of tetraethylene glycol connected to the pyrene moiety as shown in Fig. 3. Herein, a double linker was synthesized with a terminal hydroxyl or carboxyl functional group to study the effect of these groups on the tissue formation and electrical behavior of the formed tissue. For this purpose, the reaction was begun by reacting TEG with propargyl bromide in the presence of sodium hydride to get the linker OH-TEG-alkyne (1). The synthesized linker was reacted with pyrene butyric acid through an esterification reaction using DMAP as a catalyst and EDC as a coupling agent to obtain Py-TEG-alkyne (2). On the other side, to synthesize the second linker, a selective tosylation reaction was conducted to TEG to get OH-TEG-OTs (3). Then, compound (3) was reacted with sodium azide and hence the tosyl group was replaced with azide in ethanol to get OH-TEG-N_3_ (4), as we reported previously^51^. To obtain a carboxyl-terminal, compound (4) was oxidized using Jones reagent to obtain COOH-TEG-N_3_ (5). After that, OH-TEG-N_3_ (4) or COOH-TEG-N_3_ (5) was reacted with Py-TEG-alkyne (2) through click reaction^52^. This reaction was done using sodium ascorbate and anhydrous copper sulfate as catalysts dissolved in H_2_O and DCM forming a triazole ring to synthesize Py-TEG-triazole-TEG-OH (6) or Py-TEG-triazole-TEG-COOH (7) as shown in Fig. 3.

**Figure 4:**
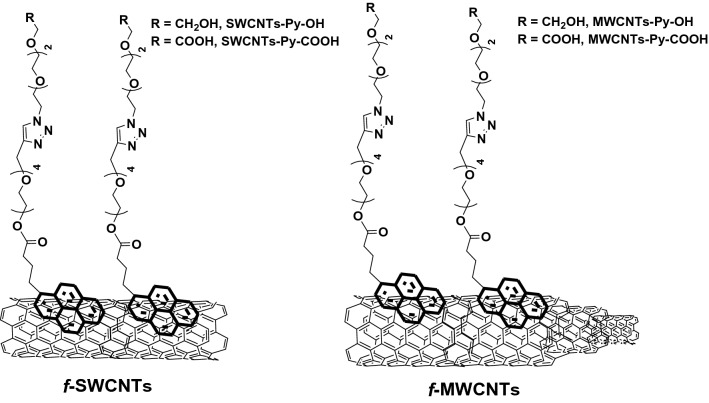
The scheme demonstrates the non-covalent functionalization of SWCNTs (left) and MWCNTs (right) with the hydroxyl group (SWCNTs-py-OH & MWCNTs-py-OH) and a carboxyl group (SWCNTs-Py-COOH & MWCNTs-Py-COOH).

Once the required compounds were successfully synthesized, their capability to functionalize and disperse the CNTs in water was investigated (Fig. 4). To optimize the amounts of the synthesized compounds (6) or (7) needed to functionalize the CNTs, various amounts were tested (0.25, 0.5, and 1 mg mL^−1^). Then, 1 mg of the CNTs was incubated with the mentioned solutions and sonicated for 30 min. After that, the dispersion and stability of the formed suspension were studied. In all cases, the best-needed amount of the Py-OH or Py-COOH was 0.5 mg mL^−1^ to functionalize 1 mg of the CNTs with good stability.

**Figure 5:**
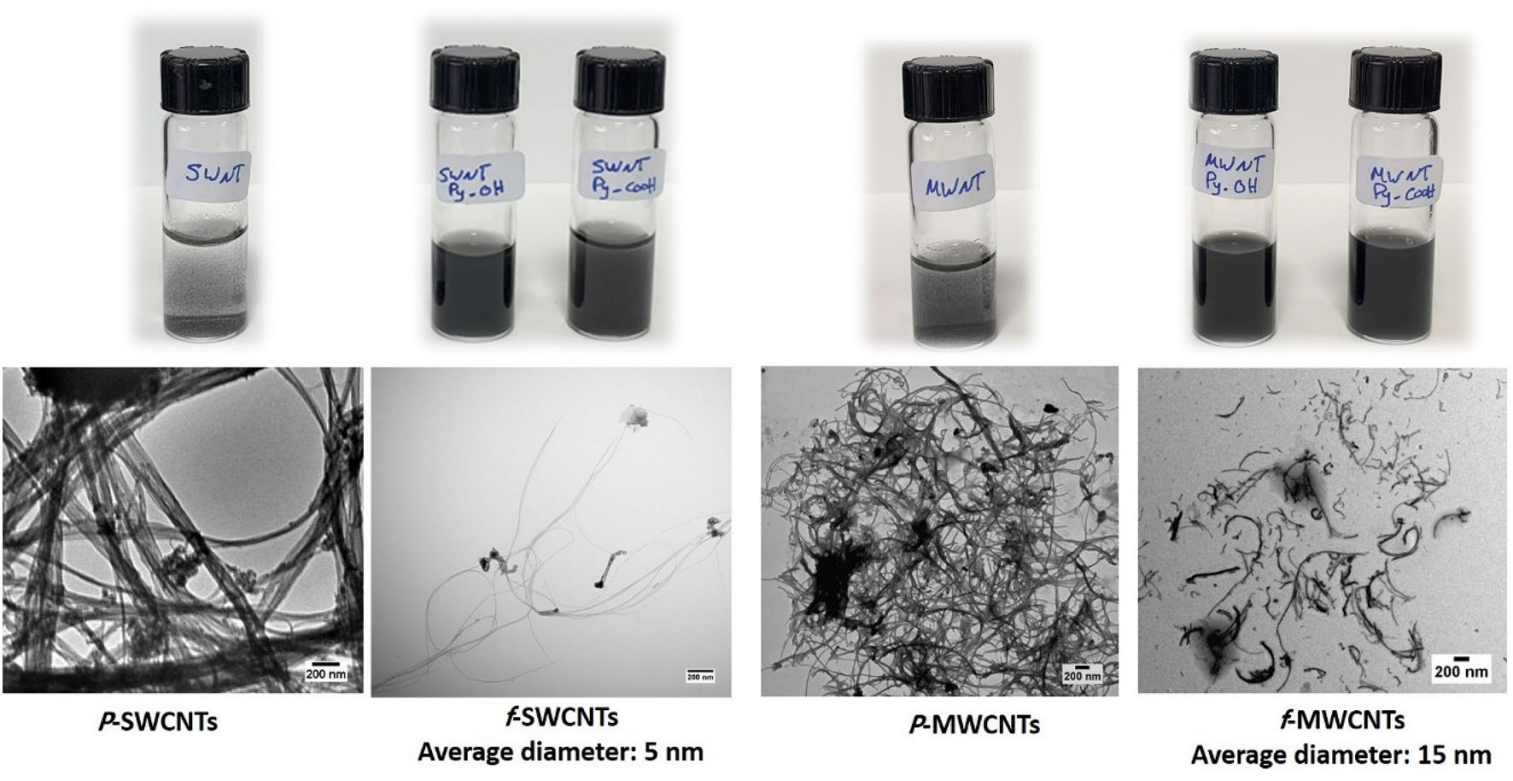
Vial photographs of pristine SWCNTs (*P*-SWCNTs); functionalized SWCNTs (SWCNTs-Py-OH and SWCNTs-Py-COOH); pristine MWCNTs (P-MWCNTs); functionalized MWCNTs (MWCNTs-Py-OH and MWCNTs-Py-COOH). TEM images of *P*-SWCNTs & *f*-SWCNTs (left image), and *P*-MWCNTs & *f*-MWCNTs (right image).

It is well-known that pristine SWCNTs and MWCNTs (*p*-SWCNTs and *p*-MWCNTs) have low water dispersibility with rapid aggregation due to their hydrophobic characteristics. Upon their functionalization with the synthesized compounds (6) and (7), the pyrene moiety interacts with the surface of the CNTs, and the double linker will be exposed to the water so increasing the hydrophilicity of the whole molecule and improving its water dispersibility as shown in the photographs in Fig. 5.

Moreover, the size and morphology of *f*-SWCNTs and *f*-MWCNTs were inspected by TEM images (Fig. 5). The TEM images of the pristine SWCNTs or MWCNTs showed the formation of huge bundles due to the Van Der Waals interactions between them. Upon the functionalization, the TEM images of *f*-SWCNTs and *f*-MWCNTs elucidate the separation between the nanotube sidewalls and so a separated nanotubes bundles with a width in the range of 5 nm for *f*-SWCNTs and 15 nm for *f*-MWCNTs can be observed (zoomed TEM images are found in Fig. S1). The functionalization of CNTs has a de-bundling effect because of the decrease in the hydrophobic interactions between the nanotube sidewalls of the *f*-SWCNTs and *f*-MWCNTs.

**Figure 6:**
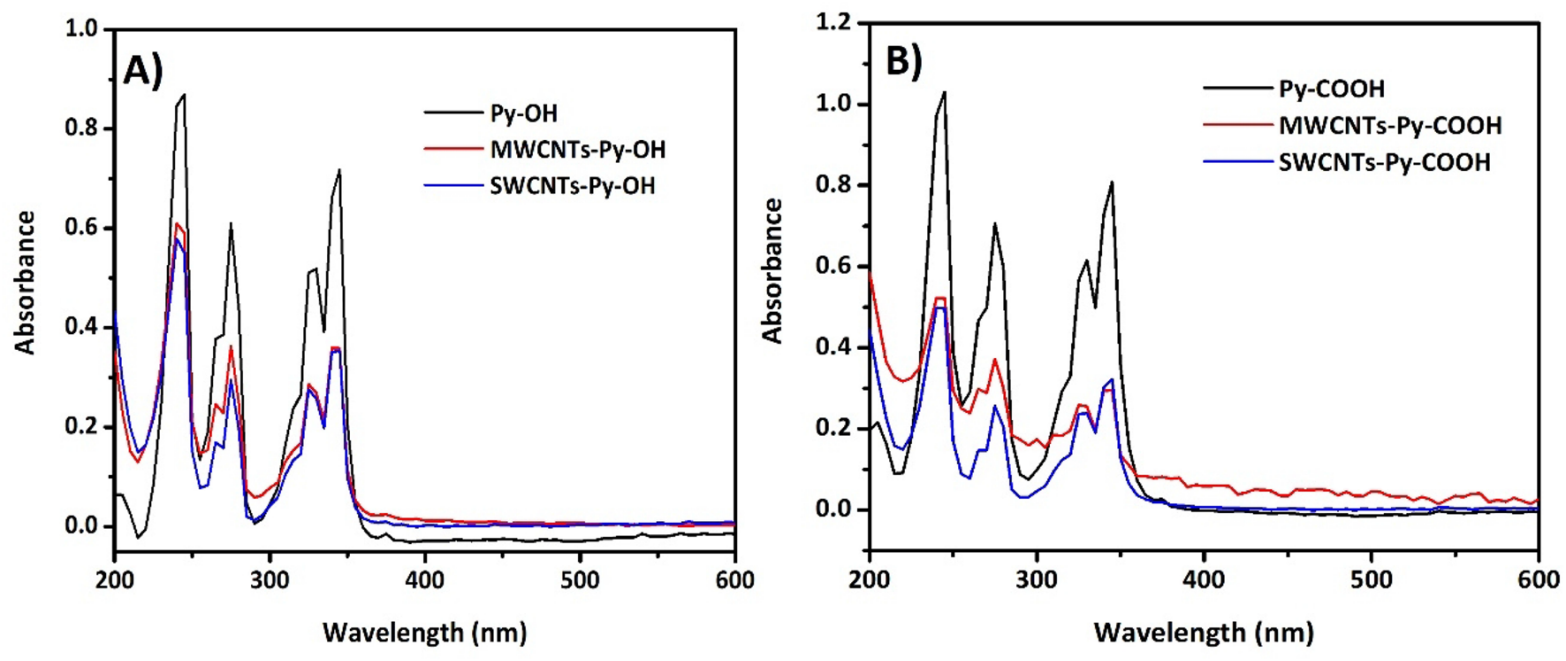
UV–Vis spectra of (**A**) Py-OH, MWCNTs-Py-OH, SWCNTs-Py-OH, and (**B**) Py-COOH, MWCNTs-Py-COOH, SWCNTs-Py-COOH.

The π-π stacking between the pyrene moieties and the CNTs was confirmed by absorption spectra. Pyrene has three characteristic peaks at 245, 275, and 345 nm due to the π-conjugation system^53^. Upon the incubation with the CNTs, the three peaks are observed which confirms the presence of pyrene moieties but there is a partial quenching effect of the absorption due to the interaction with the surface of the CNTs^54,55^. Moreover, the presence of the pyrene peaks is due to the higher molar extinction coefficient of pyrene moiety (40,000 M^−1^ cm^−1^) in comparison to that of the carbon nanotubes (4400 M^−1^ cm^−1^)^56,57^. This confirms the successful π-π stacking between the pyrene moieties and the CNTs as can be observed in Fig. 6 (UV–Vis spectra of pristine SWCNTs and MWCNTs are found in Fig. S2).

**Figure 7:**
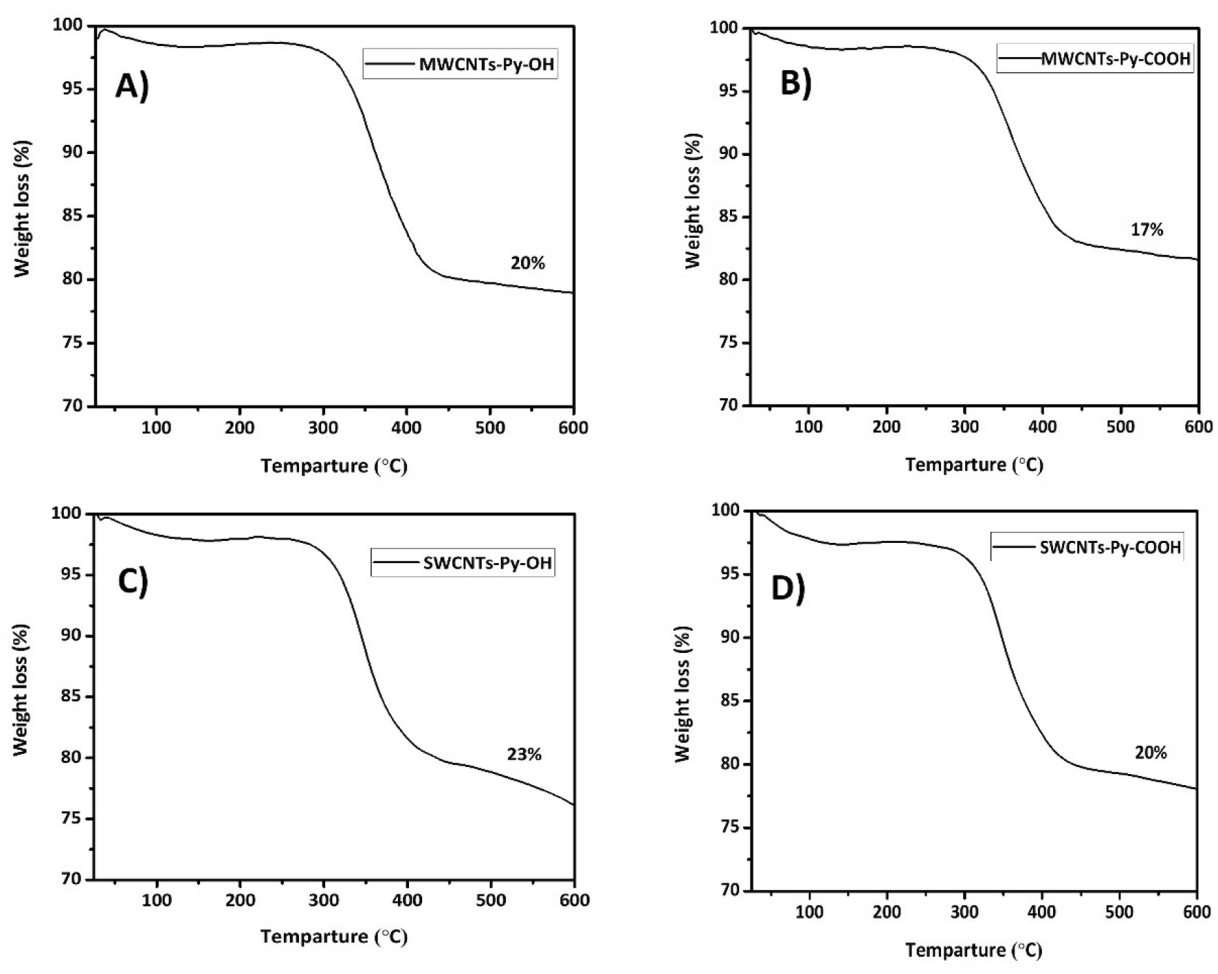
TGA analysis of all *f*-CNTs: (**A**) MWCNTs-Py-OH; (**B**) MWCNTs-Py-COOH; (**C**) SWCNTs-Py-OH and (**D**) SWCNTs-Py-COOH.

Thermogravimetric analysis (TGA) was used to quantify the amount of functionalization of the used CNTs. As shown in previous studies that the CNTs (MWCNTs, and SWCNTs) are thermostable up to 600 degrees Celsius (°C) and most organic compounds are completely degraded at this high temperature^58^. Therefore, upon heating our functionalized CNTs, the weight loss in the conjugate will be related to the number of pyrene moieties attached to the surface. In Fig. 7, it could be observed that the amount of functionalization in all CNTs is in the range of 17–23%. As the hydrophobic component is the same, therefore there is an almost equal amount of functionalization that sufficient to disperse the CNTs perfectly in the water.

Various reports have shown that dynamic light scattering (DLS) could be used to analyze the dispersity and the agglomeration behavior of the functionalized carbon nanotubes even they don’t have a spherical shape^59,60^. As can be observed in Table 1, the pristine SWCNTs and MWCNTs showed very high hydrodynamic diameter (2358 nm and 2399.1 nm, respectively) with high polydispersity index above 0.3. This due to the formation of huge agglomeration and aggregates of the pristine carbon nanotubes. However, the functionalized carbon nanotubes showed a hydrodynamic diameter around the 200 nm which is much less than the *p*-CNTs due to the dispersibility and de-aggregation of the carbon nanotubes^59^. Moreover, the polydispersity index was less than 0.3 in all *f*-CNTs which indicates the good dispersity and a the formation of a homogenous population of the functionalized carbon nanotubes^61^.

**Table 1:** Hydrodynamic diameter and zeta potential values for the pristine CNTs and our *f*-CNTs.

CNTs	Hydrodynamic diameter (nm)	Polydispersity index	Zeta potential (mV)
*P*-SWCNTs	2858.0	0.599	− 15.14 ± 0.5
SWCNTs-Py-OH	207.6	0.283	− 19.41 ± 1.0^a^
SWCNTs-Py-COOH	210.5	0.241	− 18.11 ± 1.2^a^
*P*-MWCNTs	2399.1	0.462	− 17.82 ± 1.4
MWCNTs-Py-OH	206.6	0.263	− 21.12 ± 0.2^b^
MWCNTs-Py-COOH	212.3	0.232	− 20.47 ± 0.6^b^

All experiments were conducted in triplicate. One-way ANOVA test was used to compare the means.

^a^P value < 0.05 comapre to the *P*-SWCNTs.

^b^P value < 0.05 comapre to the *P*-MWCNTs.

Moreover, zeta potential is used to analyze the charge of the outer surface of the nanomaterials. Studies showed that a zeta potential value above ± 20 mV indicates the formation of a stable suspension^62^. Herein, we measured the zeta potential for the pristine CNTs and all of our *f*-CNTs (SWCNTs-Py-OH, SWCNTs-Py-COOH, MWCNTs-Py-OH, and MWCNTs-Py-COOH) using the phase analysis light scattering (PALS) technique (Table 1). We obtained in all *f*-CNTs negative values around − 20 mV in comparison to the pristine CNTs that indicate the formation of a stable suspension and the increase in negative values is statistically different in comparison to the pristine carbon nanotubes due to the higher presence of hydroxyl and carboxyl groups on the surface of the functionalized carbon nanostructures.

### Generation of engineered connective tissues with 3T3 cells

We have previously reported the development of engineered connective tissues based on 3T3 cells for the improvement of wound healing and the re-epithelization using chitosan-functionalized CNTs^40^. In addition, a previous study by our group demonstrated that the incorporation of CNTs in the collagen matrix of ECTs could enhance several of their biomechanical properties like tissue stiffness, elasticity, resilience, toughness, and extensibility^63^. However, the electrical conductivity of the engineered tissue is an important property that provides better tissue repair and stimulates growth and cellular activity through electrical transfer^64–66^. Herein, once the CNTs were successfully functionalized non-covalently with the proper spacers, we studied their incorporation in the tissue formation and the electrical conductivity of the engineered tissue, histopathological examination and the morphology of the formed tissue fiber were investigated.

Collagen-based ECTs containing 3T3 cells were generated with different concentrations of CNTs. The control tissues appeared as whitish, opaque, rounded, and cohesive disks while the incorporation of CNTs loading made the tissues appear dark-gray depending on the kind and concentration of CNTs. There was no contraction in the tissues over 5 days in culture as the tissues were still occupying the whole culture surface of the well in a 48-well-plate.

#### Cell viability test

**Figure 8:**
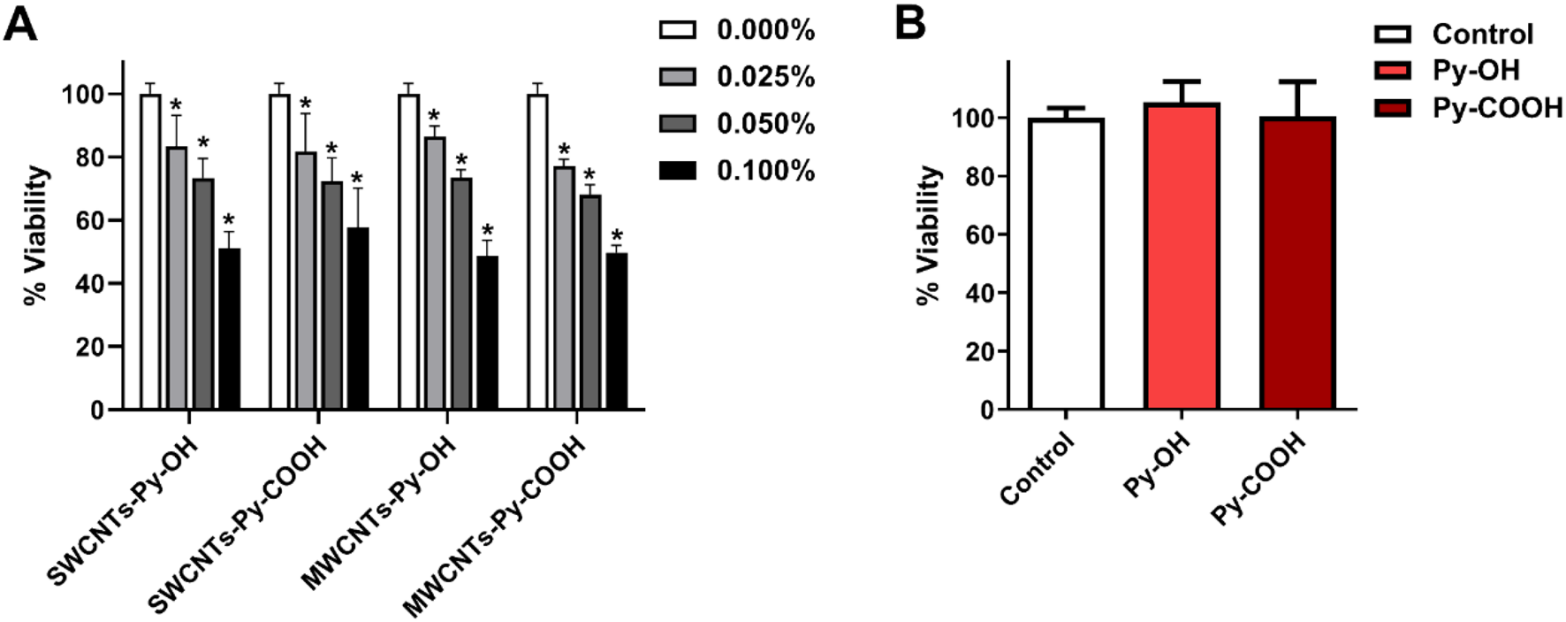
(**A**) Concentration-dependent effect of CNTs species on the viability of 3T3 cells over 24 h. (**B**) Testing the effect of 0.1% Py-OH and Py-COOH on the cell viability. The symbol * indicates significance (*P* ≤ 0.05) compared to the control (0.000%), n = 8. The statistical significance was determined by the one-way ANOVA test.

Monolayer two-dimensional cultures of 3T3 cells were incubated over 24 h in culture media containing CNTs test compounds in equivalent concentrations to those used in the ECTs. The viability of 3T3 cells was investigated by MTS assay. The data demonstrated that compared to the control cells there was a statistically significant concentration-dependent reduction in 3T3 cells viability. The concentration of 0.025% of CNTs reduced the cell viability on average by around 13–23% (Fig. 8).

#### Electrical conductivity of ECTs

**Figure 9:**
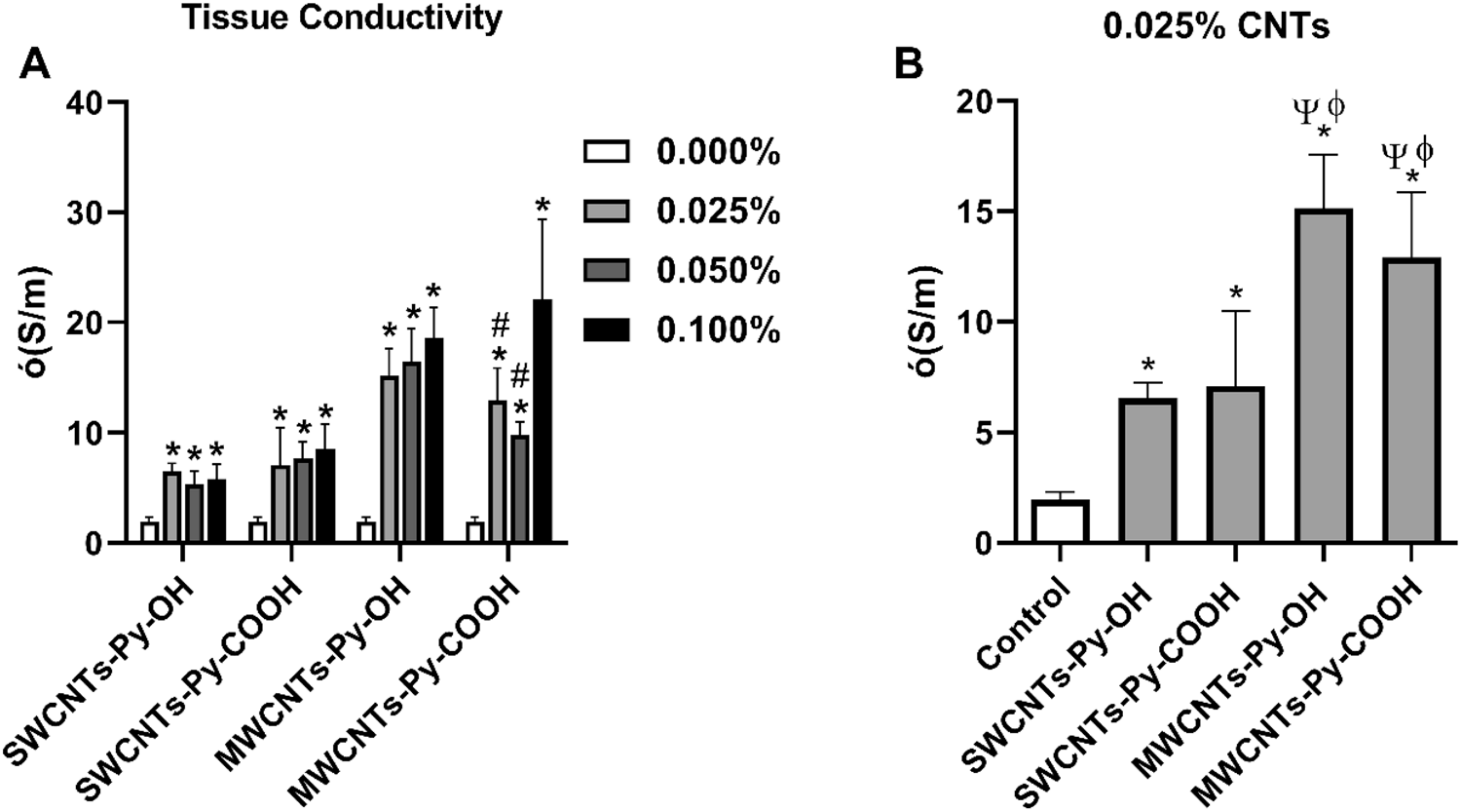
(**A**) Average electrical conductivity of collagen-based ECTs loaded with different concentrations of CNT test compounds. *P ≤ 0.05 compared to the control (0.000%) which contains only collagen in the matrix, #*P* ≤ 0.05 compared to 0.100% concentration of the test compound, (**B**) Comparison of the average electrical conductivity between the different species at 0.025% concentration of the CNTs. ψ *P* ≤ 0.05 compared to SWCNT-Py-OH, ϕ *P* ≤ 0.05 compared to SWCNT-Py-COOH as determined by one-way ANOVA test, n = 5–9.

The electrical conductivity of these tissues was measured, herein the data demonstrated that the formation of an integrated three-dimensional network of all kinds of CNTs within the collagen matrix was associated with a significant enhancement in the electrical conductivity that was mostly kind-dependent (Fig. 9A).

Various studies showed that the MWCNTs and SWCNTs have an almost comparable electrical conductivity^67–69^. But, in our research, we found that the *f*-MWCNTs have a higher electrical conductivity that could be attributed to the better dispersibility, and homogeneity in the engineered tissue. In addition, as the MWCNTs have multi enrolled layers of graphene, the functionalization will be only in the outer layer of the nanotube and keeping the inner layers intact and permitting the continuous electron flow^70^. The data demonstrated that the enhancement of the conductivity was highest with MWCNTs-Py-COOH and MWCNTs-Py-OH compared to SWCNTs-Py-COOH, and SWCNTs-Py-OH (Fig. 9A). Therefore, in general, MWCNTs species can enhance the conductivity more than SWCNTs species do (Fig. 9B). In addition, except for MWCNTs-Py-COOH, the increase of the CNTs species beyond 0.025% did not result in a statistically significant increase in tissue conductivity, and this concentration was already enough to significantly enhance the electrical conductivity of the tissues with an acceptable degree of cytotoxicity as shown before in Fig. 4. Therefore, we believe that there would be no need to include the higher concentrations in future studies for generating electrically conductive tissues. It is noteworthy that the increase in tissue conductivity observed above (Fig. 9) was not affected by the reduction in cell viability, indicating that the tissue conductivity is mainly dependent on the composition of the matrix, rather than the cell content.

#### Histopathological evaluation for ECTs

**Figure 10:**
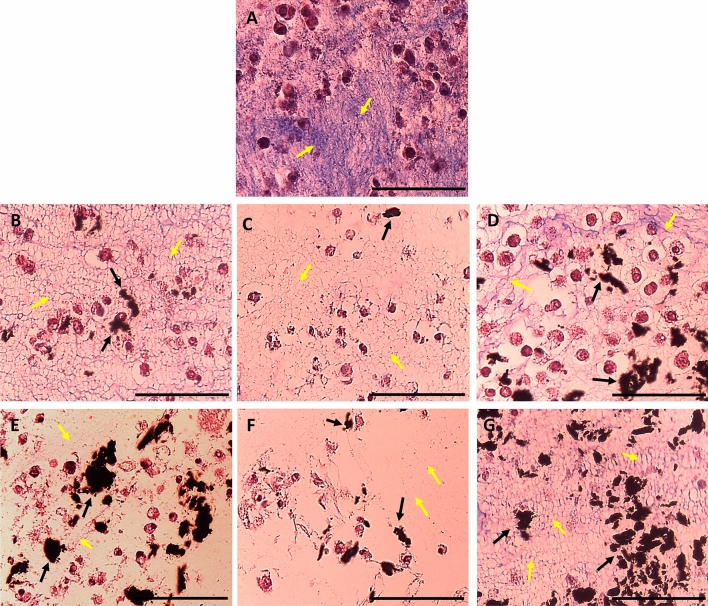
Microscopic images of collagen-based ECTs containing different concentrations of SWCNTs-Py-OH or SWCNT-Py-COOH. The tissue sections were stained with Masson’s trichrome stain and examined with a light compound microscope. (**A**) Control ECT; (**B**) 0.025% SWCNTs-Py-OH; (**C**) 0.050% SWCNTs-Py-OH; (**D**) 0.100% SWCNTs-Py-OH; (**E**) 0.025% SWCNTs-Py-COOH; (**F**) 0.050% SWCNTs-Py-COOH; (**G**) 0.100% SWCNTs-Py-COOH. Scale bar 100 µm.

**Figure 11:**
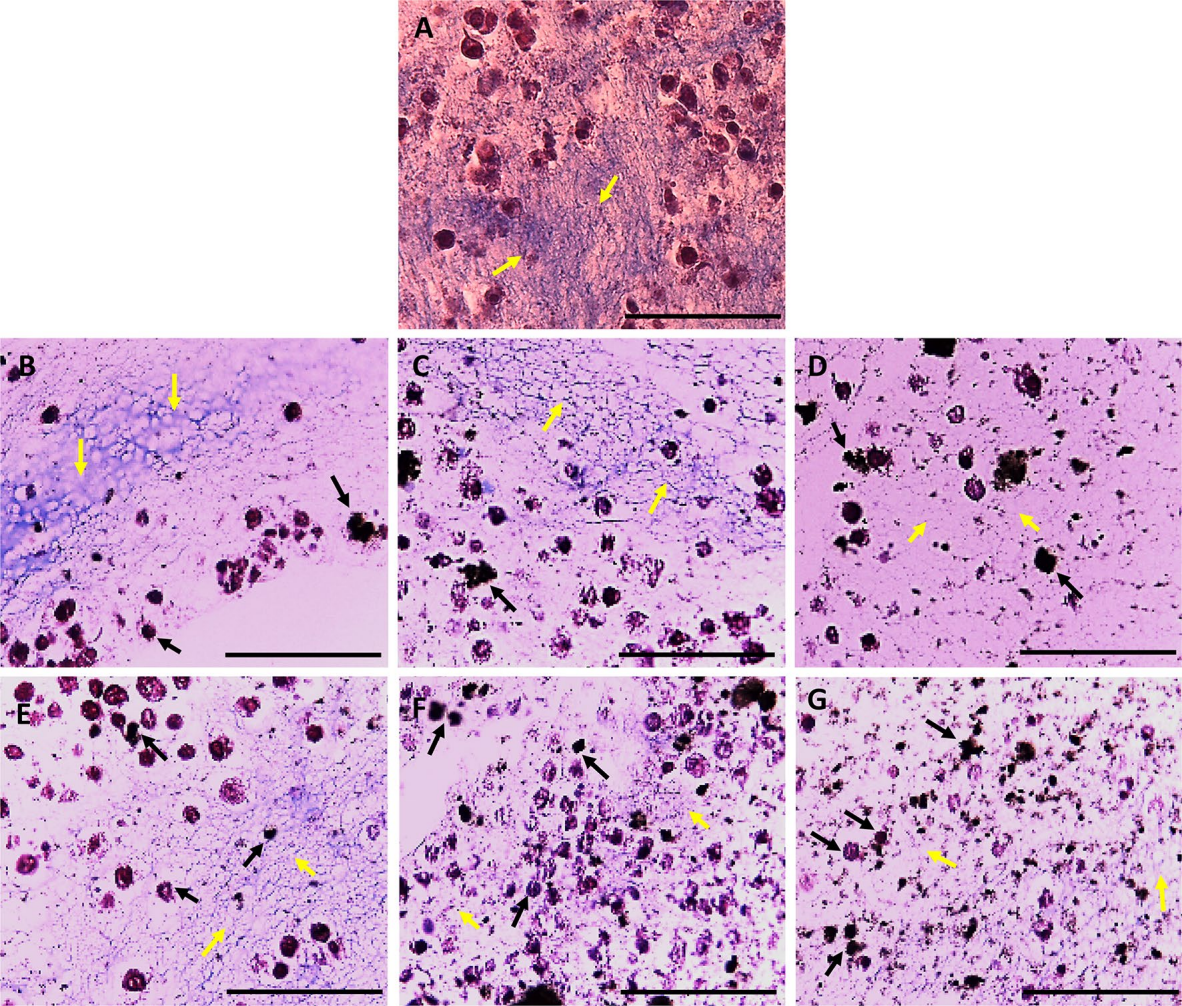
Microscopic images of collagen-based ECTs containing different concentrations of MWCNTs-Py-OH or MWCNT-Py-COOH. The tissue sections were stained with Masson’s trichrome stain and examined with a light compound microscope. (**A**) Control ECT; (**B**) 0.025% MWCNTs-Py-OH; (**C**) 0.050% MWCNTs-Py-OH; (**D**) 0.100% MWCNTs-Py-OH; (**E**) 0.025% MWCNTs-Py-COOH; (**F**) 0.050% MWCNTs-Py-COOH; (**G**) 0.100% MWCNTs-Py-COOH. Scale bar 100 µm.

Masson’s trichrome stain was utilized to study the level of fibrosis, the organization of ECM, morphology, and distribution of 3T3 cells within the tissues, and possible interaction of the CNTs with the cells. Despite the improved dispersibility of SWCNTs and MWCNTs in an aqueous solution after functionalization with polar moieties (as shown in Fig. 5), the functionalized SWCNTs and MWCNTs tended to form some tiny aggregates when mixed with the liquid collagen solution during the casting process, which could be observed later on in the histology slides (Figs. 10 and 11). This could be attributed to the high viscosity of the collagen solution (before collagen polymerization during the casting of the tissues), which could have packed some CNT particles together forming small lumps. It is worth mentioning that it was not technically possible to treat the mixture vigorously to disintegrate these aggregates so that no air bubbles are trapped within the viscous collagen solution, which could drastically deteriorate the quality of the matrix. However, we hypothesize that there was enough amount of the dispersed CNTs within the tissues (as evident by the significant improvement in the tissue conductivity shown in Fig. 9), but this could not be visualized by the compound light microscope due to the tiny diameter of the CNTs, which is within the nanometer scale as mentioned before.

A blinded-histopathological evaluation of the tissues demonstrated that the ECM of all tissues was disorganized (yellow arrows in Figs. 10 and 11) indicating that the cells were not able to organize the matrix (Fig. 10) Normally fibroblasts tend to organize the collagen fibers along with stress directions. In our tissues, there was no directional stress imposed on the tissues^71^, therefore, the collagen fibers were not oriented and appeared disorganized.

Aggregates of The SWCNTs-Py-OH and SWCNTs-Py-COOH were detected as a random dispersion within the ECM (Fig. 10, black arrows). However, the colocalization of the CNTs with the cells was not clear.

In the samples containing MWCNTs-Py-OH and MWCNTs-Py-COOH (Fig. 11), the CNT particles were dispersed throughout the ECM, and many of them colocalized with the cells (black arrows) at all of the tested concentrations. In the same context, with the concentrations of 0.100% and 0.050% of these two compounds (Fig. 11C,D,F,G) the ECM was highly disorganized as compared to the 0.025% concentration of the corresponding compound (Fig. 11B,E, yellow arrows,). Here the cells appeared more intact despite the colocalization of MWCNTs-Py-OH and MWCNTs-Py-COOH. This indicates that the 0.025% concentration of MWCNTs-Py-OH or MWCNTs-Py-COOH could be of low toxicity to the cells, which is in line with the viability studies by the MTS assay shown above (Fig. 8).

**Figure 12:**
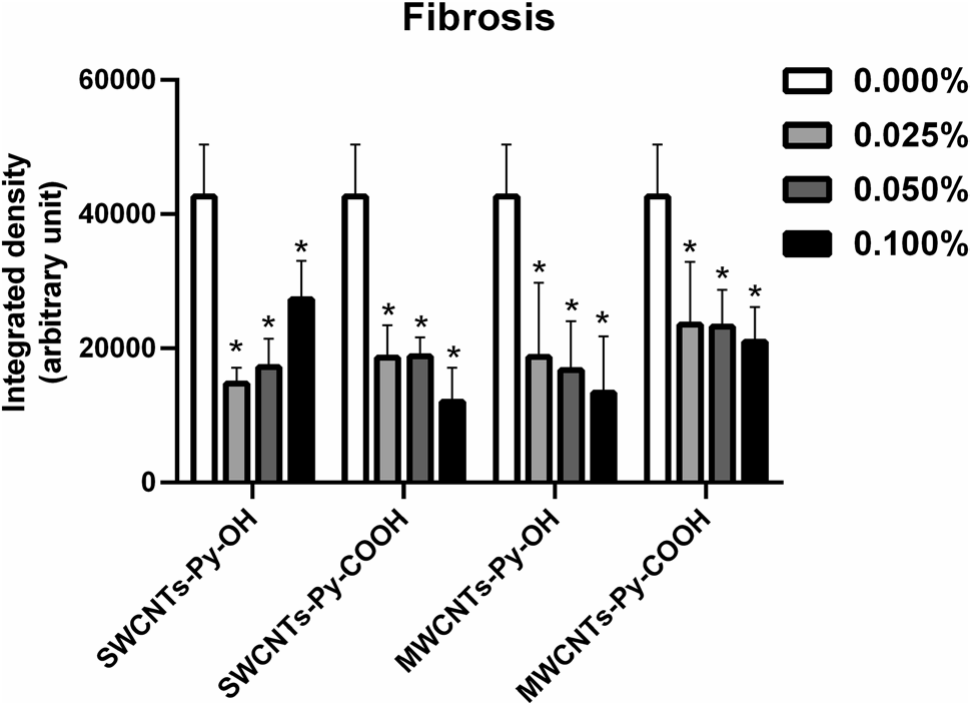
Digital analysis of the degree of fibrosis in the collagen-based ECTs. ECTs containing different concentrations of SWCNTs-Py-OH, SWCNTs-Py-COOH, MWCNTs-Py-OH, or MWCNTs-Py-COOH. *p < 0.05 compared to control (0.000%) as assessed by a one-way ANOVA test, n = 12.

Interestingly, SWCNTs-Py-OH generally demonstrated a concentration-dependent increase in tissue fibrosis despite the decrease in the viable 3T3 cells. We assume that this could be due to the induction of collagen deposition by the remaining stressed cells^40^, but this needs to be further investigated by future projects. Generally, collagen consists of three chains of helical proteins that wound around one another forming a right-handed triple helix. The three-stranded helical collagen molecules pack side-by-side together to form strong collagen fibrils, that are stabilized by covalent aldol cross-links^72^. The formation of such cross-linked collagen is enhanced during the process of tissue fibrosis^73^. In this project, the intensity of fibrosis in random images taken for all ECTs was digitally analyzed by ImageJ software. As shown in Fig. 12, all of the tested conditions significantly decreased the formation of dense collagen fibrils, i.e. prevented the formation of fibrotic tissues, which we hypothesize to be due to the interruption of aldol cross-links formation between adjacent collagen molecules. To gain a deeper insight into the structure of the ECM in our tissues, investigations by SEM were performed.

#### Scanning electron microscope evaluation for ECTs

**Figure 13:**
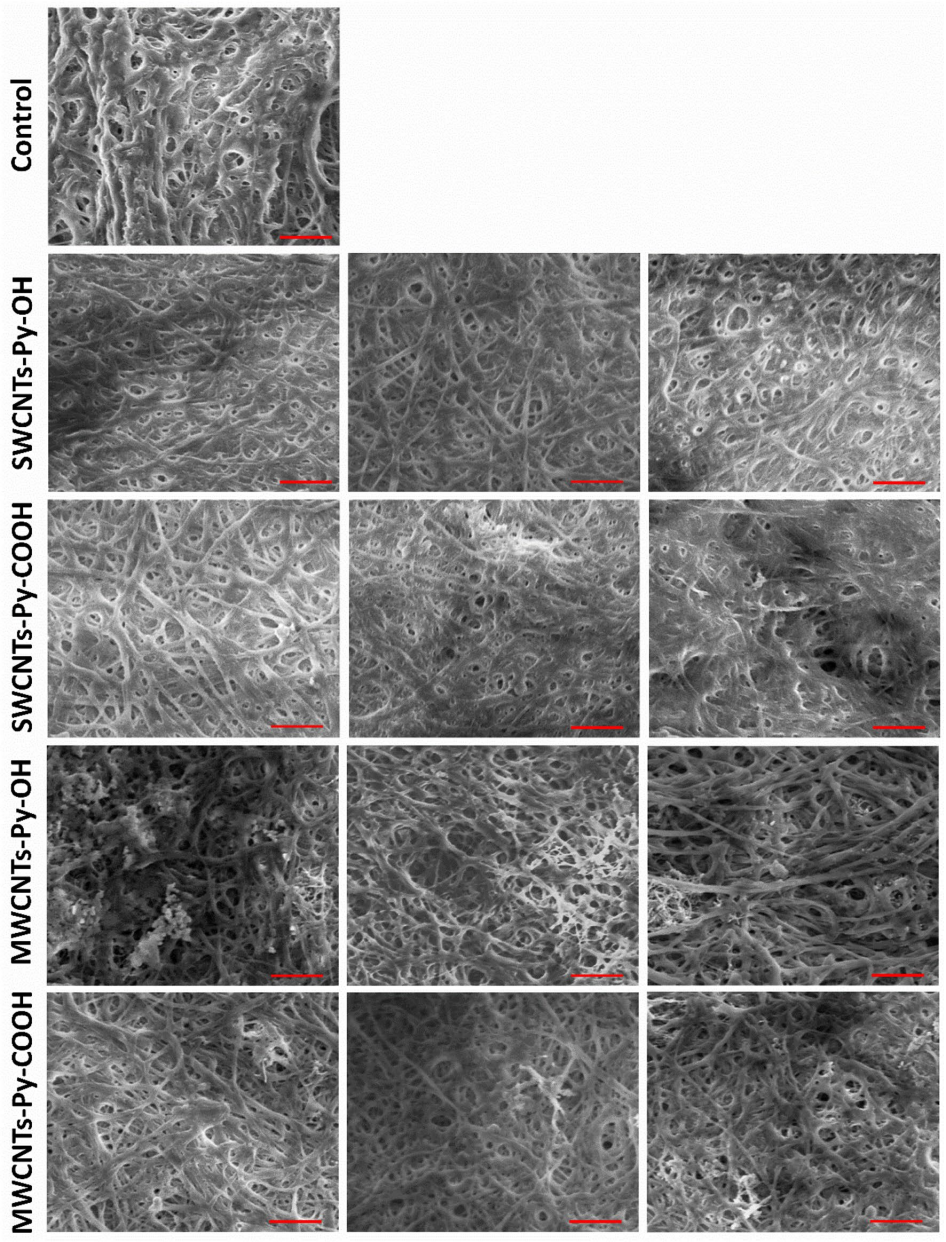
SEM images for collagen-based ECTs containing different concentrations of SWCNT and MWCNT species; Left: 0.025%, middle: 0.050%, right: 0.100%. Scale bar = 1 μm.

Detailed SEM imaging of the different kinds of collagen-based ECTs revealed the formation of distinct patterns of interweaving networks of collagen fibers and CNTs, especially in the spaces between the fibers (Fig. 13). The images were digitally analyzed by ImageJ software to highlight the differences in the microstructures of the tissue matrices as detailed below.

##### Analysis of fiber thickness

**Figure 14:**
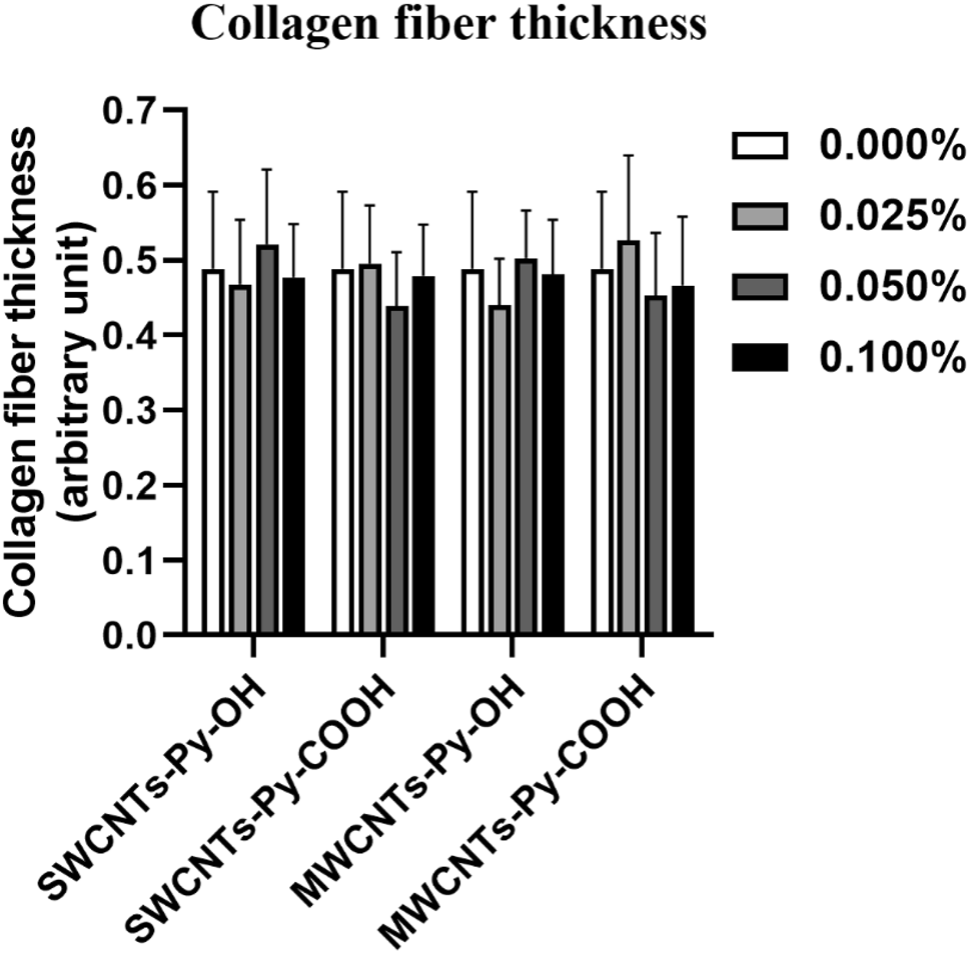
SEM evaluation of collagen-based ECT constructs of 3T3 cells with different loaded CNTs. Collagen fiber thickness was digitally quantified by ImageJ. The data were analyzed by a one-way ANOVA test, n = 12.

Digital analysis of SEM images by ImageJ software demonstrated that the thickness of collagen fibers in all conditions was similar to that of the control (Fig. 14). This indicates that the inclusion of CNTs species did not affect the polymerization of collagen fibers.

##### Analysis of matrix porosity

**Figure 15:**
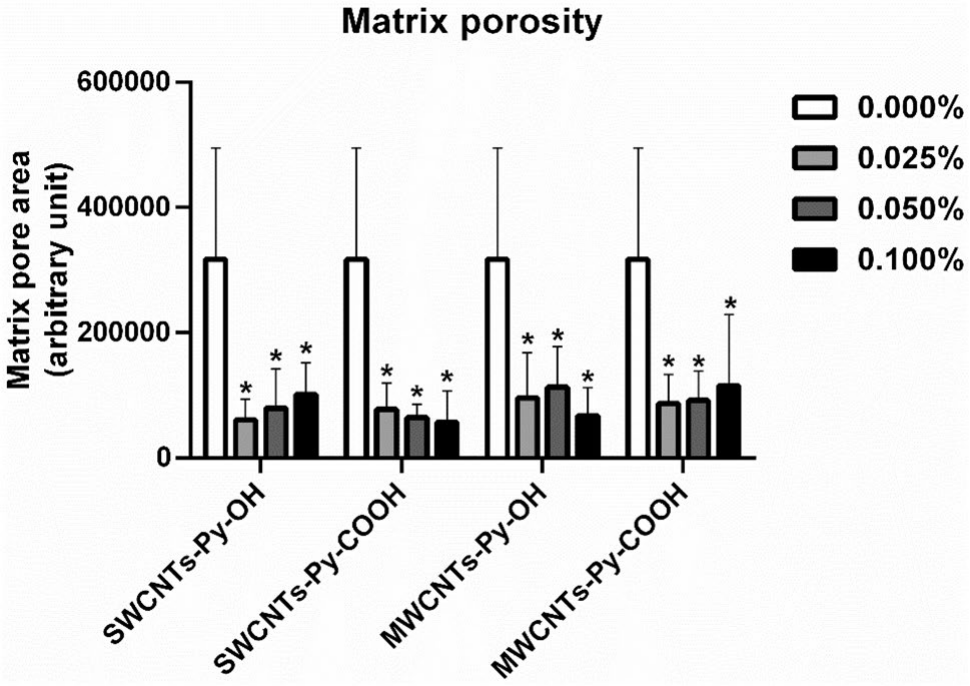
SEM evaluation analysis of collagen-based ECT constructs of 3T3 cells with different loaded CNTs. The symbol * indicates significance (*P* ≤ 0.05) compared to the control (0.000%). The statistical significance was determined by one-way ANOVA, n = 12. Matrix porosity was digitally quantified by ImageJ.

The matrix porosity of random images taken for all collagen-based ECTs was digitally analyzed by ImageJ software (Fig. 15). All CNTs-containing ECTs exhibited a statistically significant decrease in matrix porosity relative to the control. The decrease in matrix porosity could reflect the formation of more compact matrices in the presence of CNTs, which we hypothesize to result from an interaction between the collagen fibers on the one side and CNTs on the other side. The change in tissue porosity should be a subject for future in-depth analysis to elucidate its potential effect on the physical properties of the tissues.

Interestingly, in the current work, the integration of the *f*-CNTs in the matrix of the engineered connective tissue enhances the electrical conductivity with acceptable cell viability of the 3T3 cells at low concentrations. Moreover, the developed ECTs have decreased the formation of fibrosis in comparison to the control with the reduction of the matrix porosity. To our knowledge, there are no previous studies that functionalized the SWCNTs and MWCNTs non-covalently with pyrene-linker moiety and developed an engineered tissue based on the 3T3 cells. However, other previous research works used different types of functionalization with different cell lines. MacDonald et al*.* developed an engineered tissue based on SWCNTs, collagen type I, and human dermal fibroblast cells (HDF), the loaded amount of the SWCNTs was 0.8, 2.0, and 4.0 wt.%. The viability of the HDF in the constructs showed high viability and an increase in the electrical conductivity (3–7 mS cm^−1^) upon increasing the %weight of the SWCNTs^74^. Another study conducted by Voge et al*.* that they have utilized carboxylated MWCNTs to be wrapped with either pluronic acid or gelatin to construct a 3D composite of MWCNTs-fibrin-collagen seeded with fibroblast. The results demonstrated that the MWCNTs functionalized with pluronic acid showed a good dispersibility and stability which decrease the electrical impedance by two orders in magnitude in comparison to the matrix containing pristine CNTs. The cellular activity was measured by XTT assay and the data showed that the decrease in the metabolic activity is concentration-dependent with a range of 60–90% at a concentration of 0.15 mg ml^−1^^70^. In our study, we are able to construct engineered tissues based on the non-covalently functionalized CNTs that showed high electrical conductivity of 6.51–7.09 S m^−1^ in the case of the tissue containing 0.025% of the *f*-SWCNTs and 12.90–15.15 S m^−1^ in the case of the tissue containing 0.025% of the *f*-MWCNTs.

Despite the interesting physicochemical properties of CNTs, they are not free of toxicity, which has been investigated by many researchers. These studies were reviewed by several comprehensive review articles such as those by Madani et. al., 2013 and Ema et. al. 2016. In summary, pristine CNTs (CNTs without the addition of any functional group) may produce several toxic reactions depending on several factors like the dose, route of administration, exposure time, and purity. However, most of the available in vivo studies were conducted by the administration of CNTs to rodents by inhalation or intratracheal instillation, which demonstrated significant pulmonary toxicity. Systemic administration of pristine CNTs showed an accumulation of CNTs in the liver, spleen, heart, and lungs. The long-term accumulation in these organs resulted in low-grade toxicities that were mediated by oxidative stress and inflammation. Interestingly, the functionalization of the pristine CNTs may significantly improve the dispersibility of CNTs in aqueous environments and may also greatly reduce their toxicity^75,76^. This approach was taken into account in this project. we hypothesize that even if our ECTs would be implanted in an animal model in the future the distribution of CNTs from the implants to the systemic circulation might be at a low rate, which might not permit toxic plasma levels. This hypothesis has to be investigated in detail in future work.

## Conclusion

Effective noncovalent functionalization of CNTs has been achieved, which provided a stable dispersion of these macromolecules in water. The collagen-based ECTs containing 3T3 cells demonstrated a significant enhancement in the electrical conductivity when the collagen matrix contained dispersed *f*-CNTs, and that *f*-MWCNTs were superior to the *f*-SWCNTs in this respect. This could be achieved by concentrations of *f*-CNTs with an acceptable level of toxicity. Taken together, the data derived from the developed constructs in this study could form the basis for future development of optimized excitable engineered tissues for various in vivo applications.

## Supplementary Information

Supplementary Figures.

## Data Availability

All data generated or analyzed during this study are included in this published article.
