# Supplementary material for: Noncovalent functionalization of carbon nanotubes as a scaffold for tissue engineering
Source: Sci Rep. 2022 Jul 14;12:12062. doi: 10.1038/s41598-022-16247-7 (PMC9283586; doi:10.1038/s41598-022-16247-7)
Supplement: Supplementary file 1 — Supplementary Figures. [file 41598_2022_16247_MOESM1_ESM.docx]

**Supporting information for:**

**Noncovalent Functionalization of Carbon Nanotubes as a Scaffold for Tissue Engineering**

Mohyeddin Assali,^*1^ Naim Kittana,^*2^ Sahar Alhaj-Qasem,^1^ Muna Hajjyahya,^3^ Hanood Abu-Rass,^2^ Walhan Alshaer,^4^ Rula Al-Buqain^4^


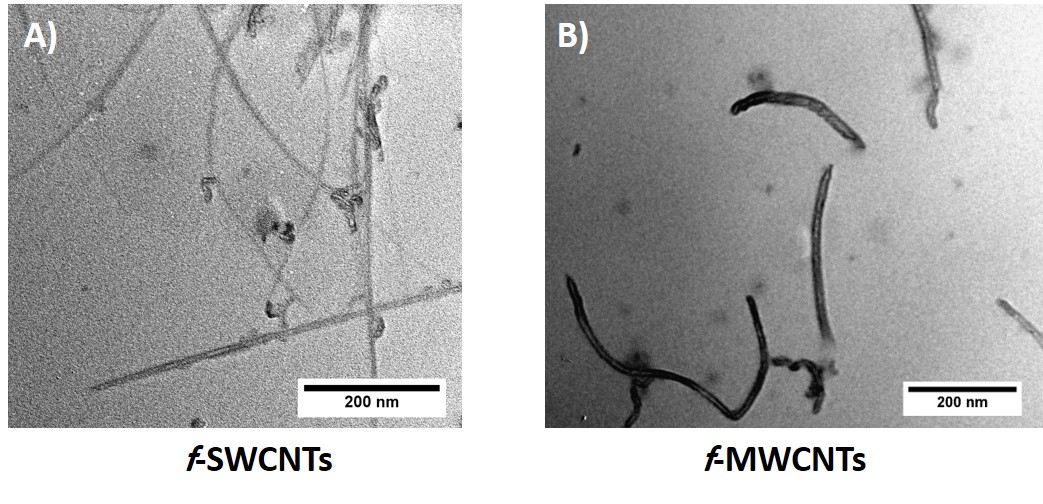


**Figure S1.** Zoom TEM images of A) functionalized SWCNTs and B) functionalized MWCNTs.





**Figure S2.** UV-vis spectra of pristine SWCNTs and pristine MWCNTs.
